# Use of patient-derived explants as a preclinical model for precision medicine in colorectal cancer: A scoping review

**DOI:** 10.1007/s00423-023-03133-7

**Published:** 2023-10-10

**Authors:** Milton Mui, Molly Clark, Tamara M. S. H. Vu, Nicholas Clemons, Frédéric Hollande, Sara Roth, Robert Ramsay, Michael Michael, Alexander G. Heriot, Joseph C. H. Kong

**Affiliations:** 1 Division of Cancer Surgery, Peter MacCallum Cancer Centre, Melbourne, VIC Australia; 2 Sir Peter MacCallum Department of Oncology, The University of Melbourne, Melbourne, Victoria Australia; 3 Department of Colorectal Surgery, Alfred Hospital, Melbourne, Victoria Australia; 4 Department of Clinical Pathology, The University of Melbourne, Melbourne, Victoria Australia; 5 Victorian Comprehensive Cancer Centre, The University of Melbourne Centre for Cancer Research, Melbourne, Victoria Australia; 6 Division of Medical Oncology, Peter MacCallum Cancer Centre, Melbourne, Victoria Australia

**Keywords:** Colorectal cancer, Patient-derived explants, Tumour microenvironment, Precision medicine

## Abstract

**Purpose:**

Whilst the treatment paradigm for colorectal cancer has evolved significantly over time, there is still a lack of reliable biomarkers of treatment response. Treatment decisions are based on high-risk features such as advanced TNM stage and histology. The role of the tumour microenvironment, which can influence tumour progression and treatment response, has generated considerable interest. Patient-derived explant cultures allow preservation of native tissue architecture and tumour microenvironment. The aim of the scoping review is to evaluate the utility of patient-derived explant cultures as a preclinical model in colorectal cancer.

**Methods:**

A search was conducted using Ovid MEDLINE, EMBASE, Web of Science, and Cochrane databases from start of database records to September 1, 2022. We included all peer-reviewed human studies in English language which used patient-derived explants as a preclinical model in primary colorectal cancer. Eligible studies were grouped into the following categories: assessing model feasibility; exploring tumour microenvironment; assessing ex vivo drug responses; discovering and validating biomarkers.

**Results:**

A total of 60 studies were eligible. Fourteen studies demonstrated feasibility of using patient-derived explants as a preclinical model. Ten studies explored the tumour microenvironment. Thirty-eight studies assessed ex vivo drug responses of chemotherapy agents and targeted therapies. Twenty-four studies identified potential biomarkers of treatment response.

**Conclusions:**

Given the preservation of tumour microenvironment and tumour heterogeneity, patient-derived explants has the potential to identify reliable biomarkers, treatment resistance mechanisms, and novel therapeutic agents. Further validation studies are required to characterise, refine and standardise this preclinical model before it can become a part of precision medicine in colorectal cancer.

**Supplementary Information:**

The online version contains supplementary material available at 10.1007/s00423-023-03133-7.

## Introduction

The management of colorectal cancer (CRC) has evolved over time but still involves a combination of surgery, chemotherapy and radiotherapy. More recently, targeted therapies and immunotherapies have become powerful additions to the therapeutic armoury against CRC, with the aim of moving us closer towards precision medicine. However, there are still issues that need to be addressed before it becomes a reality, such as identifying predictive biomarkers of treatment response beyond mutational status (e.g. MMR, RAF/BRAF) to allow patient stratification, understanding mechanisms for treatment response and resistance, and developing novel strategies for poor treatment responders.

For locally advanced rectal cancer, the current recommended treatment is neoadjuvant chemoradiotherapy (NACRT) followed by surgery. Although 10–20% of patients achieve pathological complete response, there remains a significant proportion of patients who will only partially respond or do not respond at all to current NACRT regimens [1]. Currently, all patients undergo the same treatment based on clinical and radiological staging. However, complete responders may benefit from the shift towards organ preservation and “watch and wait" strategy after NACRT to avoid overtreatment [2]. Conversely, poor responders may avoid toxicities and side effects of unnecessary treatment or benefit from alternative treatments such as total neoadjuvant therapy or immunotherapies [3–5]. In colon cancer, there is similar uncertainty in the use of adjuvant chemotherapy in patients with early (stage II) disease, with no greater than 5% benefit in 5-year disease-free and overall survival for most patients [6]. Treatment decisions have been based on high-risk features such as T4 primary, evidence of obstruction/perforation, perineural or lymphovascular invasion, and poorly differentiated histology, although the use of circulating tumour DNA has shown promising results in guiding adjuvant therapy [7].

Preclinical models such as cell lines have been used to study tumour cell biology and assess drug efficacy for many years but they lack modelling of the tumour microenvironment (TME). The role of TME, which includes immune cells, stromal cells, extracellular matrix and signalling molecules, has attracted considerable attention in recent times [8]. Indeed, the cellular composition and complex cell–cell interactions within the TME, not just tumour cell intrinsic factors, have a strong influence on tumour progression and treatment response. For example, higher levels of CD3 + and CD8 + tumour-infiltrating lymphocytes are associated with a favourable response to NACRT in rectal cancer [9], whilst regulatory T cells suppress anti-tumour immunity and are associated with poor response to NACRT [10]. In colon cancer, the introduction of the Immunoscore, an immunohistochemistry (IHC) and digital pathology-based assay which quantifies CD3 + and CD8 + T cells in the tumour core and invasive margin, has been shown to predict patients who will benefit from adjuvant chemotherapy [11]. In addition, the characteristics of the TME are altered by tumour cell metabolism that ultimately leads to chemoresistance and tumour progression. Certain conditions, such as hypoxia and acidity, have been shown to impair the function of immune effector cells and promote the accumulation of immunosuppressive cells and cytokines [12].

Therefore, a better understanding of the TME, through the use of patient-derived explants (PDEs), may be key to the discovery of more reliable biomarkers of treatment response, potential treatment resistance mechanisms, and novel treatment combinations. PDEs are obtained by cutting fresh human tumour specimens into smaller pieces and are cultured ex vivo over a period of time. The main advantage of PDEs is the preservation of native tissue architecture, TME, heterotypic cell–cell interactions and metabolic crosstalk, without excessive manipulation so they are more likely to mimic the in vivo situation [13]. In addition, PDEs are relatively easy to establish, cost-effective, and provide results in a timely manner, which may make PDEs a more robust preclinical model for translation into clinical practice. This scoping review aims to evaluate the utility of PDEs as a preclinical model in primary CRC to discover and validate potential biomarkers, explore the TME and assess drug responses in order to guide precision medicine.

## Methods

### Search strategy

This scoping review was designed and performed in accordance with the Preferred Reporting Items for Systematic Reviews and Meta-Analyses extension for Scoping Reviews (PRISMA-ScR) guidelines. A comprehensive search was conducted using the Ovid MEDLINE, EMBASE, Web of Science, and Cochrane Library databases from start of database records to September 1, 2022. The following terms and their variations were used either alone or in combination: “colorectal cancer” and “explants”. The search strategy is supplied in *Supplementary Information*. Pertinent and electronic links were hand-searched, and cross-referencing was performed for selected studies. The search results were pooled using the Covidence online platform.

### Study selection

The study included only full-text English studies including primary CRC PDEs as a preclinical model. The study excluded reviews, commentaries, posters, conference abstracts/proceedings, and studies not performed with humans. Two reviewers (M.Mui and M.C.) performed the search and extracted data independently. Eligible studies were categorized into different groups determined by the use of PDEs for assessing feasibility, exploring TME, drug testing, and biomarker discovery and validation.

## Results

**Fig. 1:**
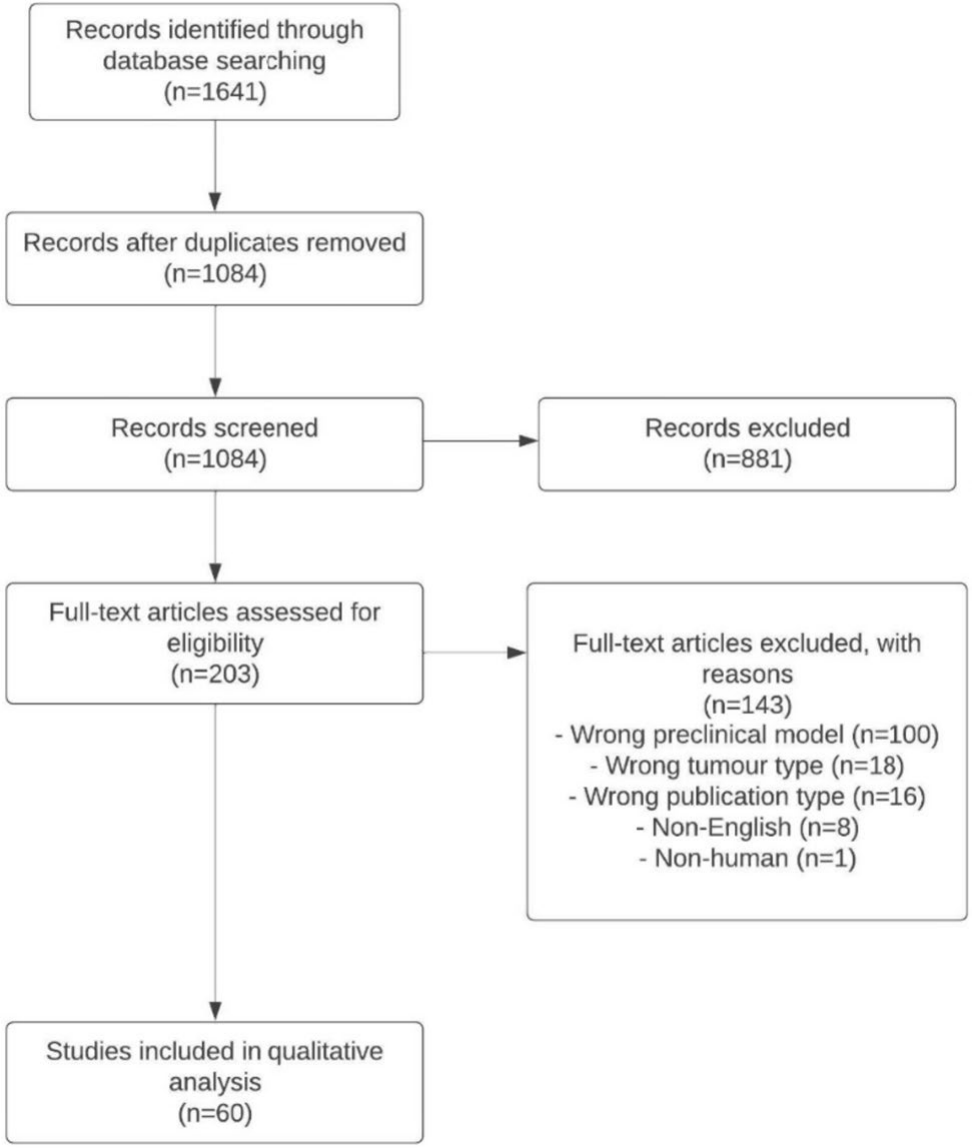
PRISMA flow diagram

The initial database search identified 1641 records, of which 203 studies were assessed for eligibility after eliminating duplicates and screening abstracts. A total of 60 studies were included in the final analysis. The PRISMA flow diagram is shown in Fig. 1. The publication years ranged from 1962 to 2022. We identified 14 studies that assessed the feasibility of using PDEs as a preclinical model for CRC, 10 studies that explored the TME, 38 studies that assessed ex vivo drug responses, and 24 studies that identified potential biomarkers of treatment response or clinical outcome.

### CRC PDE culture techniques

A variety of different culture methods and conditions were used to establish CRC PDEs as shown in Supplementary Table 1. All studies obtained tumour tissue from surgical specimens, except for one which collected biopsies endoscopically before surgery [14] and one which did not specify tissue source [15]. Tumour tissue was either processed by mechanical fragmentation or slicing using an instrument such as vibratome or tissue chopper. Subsequently, the explants were maintained either as free-floating cultures or placed on scaffolds such as gelatin or collagen sponges [14, 16–35], metal grids [36–43], or pore membranes [44–48], with or without agitation. Culture media formulations vary widely among studies but common base media include RPMI 1640 and Dulbecco's Modified Eagle Medium (DMEM), which are often supplemented with foetal bovine serum (FBS) and antibiotics. For prolonged incubation, the majority of studies used a temperature of 37°C, with 5% CO2 and 95% air, although a number of studies opted to use a higher O2 concentration [15, 37–43, 45, 47, 49, 50]. Culture duration ranged from 4 h to 122 days, depending on the aims of the study and assays performed.

### Feasibility studies on CRC PDEs

We identified 14 studies that assessed the feasibility of using PDEs as a preclinical model for CRC (Table 1). Ten studies demonstrated preservation of tissue morphology in short-term cultures as assessed by haematoxylin and eosin (H&E) [36–38, 43, 44, 46, 51–54]. Five studies performed IHC to measure proliferation markers such as Ki-67 [44, 46, 53, 55] and bromodeoxyuridine (BrdU) [52] to confirm tissue viability, although one study reported a slight but significant decrease in proliferation index after 7 days [55]. Two studies analysed gene expression patterns and found that they were relatively stable over time and comparable to the original tumour [44, 56]. One study confirmed tissue viability by demonstrating the ability to incorporate uridine and thymidine during DNA synthesis using autoradiography [49]. One study investigated the feasibility of combining proteomics using reverse-phase protein microarrays with ex vivo cultures and demonstrated the sensitivity and robustness of the system [26]. A more recent study utilised fluorescence-based imaging to assess (i) cell viability, which remained high during culture and (ii) metabolic activity, which showed a 50% decrease in the first week but remained relatively stable during the remaining culture period, suggesting a period of adaptation ex vivo [54].

**Table 1:** Feasibility studies on CRC PDEs (n = 14)

Author (year)	Assay(s) performed	Results
Rovin (1962) [36]	• H&E	• All successful cultures demonstrated maintenance of cell detail and morphology • A 5% level of CO2 was necessary for survival • Many neoplasms were well maintained in an O2 level of 1%
Wolberg (1962) [49]	• Autoradiography (for DNA synthesis)	• Tissue viability, tested by ability to incorporate uridine and thymidine, was maintained in most cases for at least 24 h of preincubation, and in some instances for as long as 48 h
Roller (1966) [37]	• H&E	• There was an overall good plus fair viability of 43%; 25% had good viability after 4 days, 14% after 7 days and 8% after 11 days • Viable specimens retained histologic characteristics of original tumours
Kalus (1972) [51]	• H&E	• Adenocarcinoma in tissue culture grew on surface of explant and invaded fibrin foam • Cytological and histological features of growing cells generally reflected pattern and cytology of parental fragments and biopsy
Pritchett (1982) [38]	• H&E	• Cell birth rate of tumour cells was 10.21 cells/1000 cells per hr compared with 7.73 cells/1000 cells per hr for mucosa
Hood (1998) [52]	• H&E • IHC (BrdU)	• There was good morphological preservation and continued proliferation for up to 7 days
Pirnia (2009) [26]	• Reverse-phase protein microarray	• Ex vivo culture system was sufficiently stable with respect to culture conditions and biologically ‘‘silent’’ with respect to expression patterns of apoptosis-related protein parameters that were investigated
Vaira (2010) [44]	• H&E • IHC (Ki-67, Akt and S6RP) • MTT • TUNEL • RT-PCR (PI3K, AKT1, S6RP) • Gene expression analysis	• Culture model preserved tissue 3D architecture, cell viability, pathway activity, and global gene expression profiles up to 5 days ex vivo
Brouquet (2011) [55]	• IHC (Ki-67)	• PolyHEMA allowed three-dimensional culture of tumour fragments up to 7 days without fibroblastic invasion and with a slight but significant decrease of proliferative index
Majumder (2015) [53]	• H&E • IHC (Ki-67) • CCK-8 assay	• Explants cultured in matched tumour-stromal matrix proteins retained tumour morphology, viability and proliferation status similar to baseline parameters
Unger (2015) [56]	• Gene expression analysis	• Patterns of freshly prepared tissue slices were closely located with those of original tumour tissue depicting similarities in gene expression • Gene expression patterns changed only marginal during short time cultivation of up to 72 h in tissue slices
Sonnichsen (2018) [46]	• H&E • IHC (Ki-67)	• Slice cultures showed good preservation of morphological features of original tumour
Da Mata (2021) [54]	• Fluorescent live/dead assay (FDA and PI) • Resazurin reduction capacity (PrestoBlue Cell Viability Reagent) • H&E • IHC (p53, MMR) • PCR (MSI, KRAS, BRAF)	• CRC PDEs retained key molecular and histological features of original tumour and partially preserved TME components
Gavert (2022) [43]	• H&E	• Ex vivo tissue culture method maintained tissue viability for at least 6 days

*H&E* haematoxylin and eosin; *IHC* immunohistochemistry; *BrdU* bromodeoxyuridine; *Akt* protein kinase B; *S6RP* S6 ribosomal protein; *MTT* 3−(4,5−dimethylthiazol−2−yl)−2,5−diphenyltetrazolium bromide; *TUNEL* terminal deoxynucleotidyl transferase dUTP nick end labelling; *RT−PCR* reverse transcription−polymerase chain reaction; *PI3K* phosphatidylinositol−3−kinase; *AKT1* AKT Serine/Threonine Kinase 1; *CCK−8* cell counting kit−8; *FDA* fluorescein diacetate; *PI* propidium iodide; *MMR* mismatch repair; *MSI* microsatellite instability; *KRAS* Kirsten rat sarcoma viral oncogene homolog; *BRAF* v−Raf murine sarcoma viral oncogene homolog B

### Exploring TME using CRC PDEs

We identified 10 studies that explored the TME using CRC PDEs (Table 2). Early studies investigated the role of enzymes such as plasminogen activator urokinase and gelatinase in CRC development and metastasis [57, 58]. The majority of subsequent studies focused on the role of different types and levels of cytokines (e.g. IL-6, IL-8, IL-10) or chemokines (e.g. CCL2, CXCL1, CXCL5, CCL5, CXCL10) in the TME that might affect cell function and migration [15, 59–63]. One study found that tumour-infiltrating T cells were able to survive in culture despite the absence of in vivo factors, suggesting that the TME may play a role [39]. A more recent study demonstrated that a high density of tumour-infiltrating lymphocytes with positive T-bet transcription factor (Tbet + TILs) was associated with higher interferon-gamma (IFN-γ) levels both at baseline and following programmed cell death protein 1 (PD-1) blockade [50].

**Table 2:** Exploring TME using CRC PDEs (n = 10)

Author (year)	Assay(s) performed	TME component(s)	Results
Harvey (1988) [57]	• Western blot • ELISA • Azocaseinolysis • Spectrozyme-UK assay	• Urokinase (plasminogen activator)	• Urokinase secretion by metastatic tumours was greatly reduced in comparison with primary tumours
Yamagata (1991) [58]	• Zymography	• Gelatinase	• All carcinoma tissue culture supernatants had an active form of gelatinase which was not detectable in normal tissue
Golby (2002) [39]	• IHC	• CD3 + T cells	• CD3 + T cells were proliferating (at a low rate) within explants after 3 days of culture, indicating that they may be sustained by factors present in tumour microenvironment
Michielsen (2011) [59]	• ELISA	• Chemokines	• Tumour conditioned media contained high levels of chemokines (CCL2, CXCL1, CXCL5) in addition to VEGF • Tumour conditioned media strongly influenced dendritic cell maturation and function
Muthuswamy (2012) [60]	• IHC • Confocal microscopy • mRNA analysis • ELISA • Chemotaxis assay	• Chemokines	• Enhanced activation of tumour-associated NF-kB by combination of IFN-a, indomethacin and poly-I:C (TLR3 ligand) selectively enhanced production of effector CD8 + T cell-recruiting chemokines (CCL5 and CXCL10) and suppressed T regulatory cell-recruiting chemokine (CCL22) in tumour tissues
O'Toole (2014) [61]	• ELISA • Cytokine Antibody Array	• Inflammatory mediators	• Tumour microenvironment of all stages of CRC contained inflammatory mediators (IL-6, IL-8, GRO, angiogenin and TIMP1) capable of suppressing local dendritic cells
Kistner (2017) [62]	• ELISA	• Chemokines	• CXCL11 secretion was significantly higher in carcinoma as compared to normal mucosa • Cytokine stimulation ex vivo led to significantly increased CXCL11 expression in tumours, but not in normal colon
Benkhelifa (2019) [63]	• ELISA • Nitric oxide level measurement	• iNOS/NO system	• Nitrite levels were nearly twice as high in metastatic CRC culture supernatants from tumour explants compared with those without metastases and healthy controls • TGFβ, CTLA-4 and IL-10 were significantly related to tumour stage progression
Ott (2019) [50]	• IHC • ELISA	• Tbet + tumour-infiltrating lymphocytes	• Density of Tbet + TILs correlated with levels of IFN-γ secreted at baseline and under PD1 blockade
Mutala (2021) [15]	• ELISA • FLICA® Caspase-1 assay	• Caspase-1/IL-18	• Tumour cells displayed an activated and functional caspase-1/IL-18 axis that contributed to drive a Th1/Tc1 response elicited by TILs expressing IL-18Ra

*ELISA* enzyme−linked immunosorbent assay; *IHC* immunohistochemistry; *CCL* C‑C motif chemokine ligand; *CXCL* C−X−C motif chemokine ligand; *VEGF* vascular endothelial growth factor; *NF−kB* nuclear factor kappa B; *IFN−a* interferon−alpha; *poly−I:C* polyinosinic:polycytidylic acid; *TLR3* toll−like receptor−3; *GRO* growth−related oncogene; *TIMP1* tissue inhibitor matrix metalloproteinase 1; *iNOS* inducible nitric oxide synthase; *TGFβ* transforming growth factor beta; *CTLA−4* cytotoxic T−lymphocyte−associated protein 4; *Tbet* T−box transcription factor; *IFN−γ* interferon−gamma;*PD1* programmed cell death protein 1; *Th1* T helper type 1; *Tc1* T cytotoxic cells, type 1

### Assessing ex vivo drug responses using CRC PDEs

We identified 38 studies that assessed ex vivo drug responses using CRC PDEs (Supplementary Table 2). Commonly used chemotherapy drugs in CRC (e.g. 5-FU, oxaliplatin, irinotecan) and their combinations were tested. More recent studies included targeted therapies such as cetuximab [29, 32, 53, 64], bevacizumab [29, 32], gefitinib [56], and selumetinib [43]. A large number of studies performed the histoculture drug response assay (HDRA) with 3-(4,5-dimethylthiazol-2-yl)-2,5-diphenyltetrazolium bromide (MTT) as an endpoint to identify effective drugs based on inhibition of tumour viability [14, 16, 17, 19, 20, 23, 24, 27–30, 32, 33, 44, 65]. Two studies performed a similar colorimetric assay using water-soluble tetrazolium 8 (WST-8) [53, 66] and one study performed a luminometric adenosine triphosphate (ATP) assay [56] to measure cell viability and assess chemosensitivity. Apart from cell proliferation assays, a number of studies compared treated and untreated explants using IHC to measure proliferation markers (e.g. BrdU [21], Ki-67 [31, 44, 46, 47, 53, 55, 64, 67], proliferating cell nuclear antigen (PCNA) [45]) or apoptotic markers (e.g. caspase-3 [31, 53, 64, 67, 68]), while other studies employed H&E staining to assess tissue morphology [38, 43, 46, 53, 54, 64, 67]. Six studies analysed drug responses on a protein and gene expression level [40, 41, 44, 45, 48, 56].

### Biomarker discovery and validation using CRC PDEs

We identified 24 studies using CRC PDEs to discover and validate potential biomarkers of treatment response or clinical outcome (Supplementary Table 3). In about half of these studies, they were correlated with treatment response using ex vivo HDRA results as a surrogate marker. Response to chemotherapeutic agents as assessed by HDRA was shown to be predictive of in vivo treatment response with moderate specificity and sensitivity [14, 17, 29, 32, 33]. One study showed that chemosensitivity to 5-FU and cisplatin depended on the presence of serum p53 antibody [18]. Another study investigated the enzyme activity of dihydropyrimidine dehydrogenase (DPD) & orotate phosphoribosyltransferase (OPRT) and found that the combination of these levels was predictive of 5-FU positive sensitivity [20]. Various immunohistochemical markers including p53 [19], p21 [19], and ATP binding cassette subfamily G member 2 (ABCG2) [65] have been correlated with response to various chemotherapeutic agents. Similarly, there were differential gene expressions of thymidylate synthase (TS) [22, 30], DPD & OPRT [25], amphiregulin (AREG) & epiregulin (EREG) [64], microRNA 34a (miR-34a) [34], and checkpoint-with-forkhead-and-ring-finger-domains (CHFR) [35] between treatment responders and non-responders. Tumours with mismatch repair (MMR) defects were closely correlated with chemosensitivities to combined regimens of PDX101 with 5-FU + leucovorin + oxaliplatin (FLOX) and 5-FU + leucovorin + irinotecan (FLIRI) [27]. A more recent study incorporated readouts from different assays (H&E, IHC and CCK-8 assay) to generate a score to predict clinical response with 91.67% specificity and 100% sensitivity [53].

## Discussion

To our knowledge, this is the first scoping review evaluating the utility of PDEs as a preclinical model in primary CRC to discover and validate potential biomarkers, explore the TME and assess drug responses. The use of PDEs is not a novel concept and has been around since the 1960s in various formats [36, 37, 49, 69–71]. Early studies investigated the feasibility of such “tissue cultures” and assessed ex vivo response to different chemotherapeutic agents. Many of these studies had small sample sizes and included different types of cancers apart from CRC, which made comparisons challenging and conclusions difficult to draw. In the 1990s, Hoffmann et al. successfully optimised and popularised the platform for drug testing, with the assay now commonly known as HDRA [72]. In addition to CRC [14, 17, 29, 32, 33], previous studies demonstrated that HDRA results correlated well with in vivo drug responses and clinical outcomes in various types of solid tumours [73–75].

Despite its initial success, PDEs have not been widely used in basic research and have had minimal impact in terms of incorporating them into cancer drug development pipeline or precision medicine approach to guide clinical decision-making. This may be due to their relatively short-term viability, low throughput due to finite amount of tissue, lack of standardised response readouts, and challenges in genetic manipulation using technologies such as siRNA or CRISPR. Preference for established preclinical models, such as cell lines and xenografts, as well as the surging popularity of organoids in the early 2000s [76] may also have played a role. Organoids are 3D, stem-cell-derived structures that resemble their in vivo tissue counterparts and can be maintained in culture, with passaging, for a potentially indefinite period. Consequently, it provides useful insights into the effects of tumour heterogeneity and allows testing of different drugs and cellular therapies, making it an attractive preclinical tool to predict therapy response. However, some of the potential disadvantages of organoid cultures include in vitro selection of specific clones that are able to grow in those conditions, lack of heterotypic cell types (e.g. stromal and immune cells), low success rates, and longer times to establish successful cultures which may limit clinical applications.

With a better appreciation of the significance of the TME in determining cancer progression and treatment response, there is renewed interest in the use of PDEs to maintain tissue architecture and reflect tumour-stromal interactions and metabolic crosstalk. In addition, they are relatively easy to establish, cost-effective, and provide results within a clinically acceptable timeframe. CRC PDEs can be generated from tumour tissue obtained from either endoscopic biopsies or surgical specimens. Because of intra-tumour heterogeneity, obtaining tissue from biopsies increases the risk of working on a sample that is not representative of the overall tumour. The risk still exists in surgical specimens, albeit lower than in endoscopic biopsies. On the other hand, obtaining tissue from surgical specimens can have the limitation of not being treatment-naïve in the case of locally advanced rectal cancer where most patients undergo NACRT before proceeding to surgery. This may alter characteristics of the original tumour and TME but also increase the risk of sampling error due to tumour regression, necrosis and fibrosis.

Different tissue processing methods have been described but mechanical fragmentation into smaller tumour pieces was most commonly performed, particularly for HDRA. Alternatively, tissue slices of varying thickness were used in some studies for better consistency and reproducibility. In contrast to the methods used in establishing cell lines and organoids, tumour fragments or slices do not need to be enzymatically digested to obtain a single cell suspension. This allows preservation of the TME containing immune cells, stromal cells and extracellular matrix that provides physical scaffolding for cellular components, as well as biochemical signals that are essential for tissue differentiation and homeostasis. Although free-floating cultures may be the simplest to establish and maintain, cells tend to migrate out of the explants to form monolayer cultures with loss of tissue architecture [77]. In order to improve nutrient and waste exchange and tissue viability, some studies have successfully incorporated agitation or adopted the use of scaffolds such as gelatin or collagen sponges into their culturing techniques.

Most contemporary studies used a commercially available cell culture medium such as RPMI 1640 or DMEM, supplemented with FBS and antibiotics. FBS has been used in human cell cultures for decades and contains a variety of growth factors that are important in overall cell proliferation and differentiation, while antibiotics reduce bacterial contamination of cultures by intestinal flora from tissue specimens. In some studies that omitted FBS, other growth-promoting supplements such as B-27 [54, 67, 68] and epidermal growth factor (EGF) [54, 56, 67, 68] were added to improve viability, although evidence is scarce and inconclusive in studies on non-CRC explants [78, 79]. With the recent development of more physiological cell culture media which mimics the composition of human plasma (e.g. Plasmax, Human Plasma-Like Medium) [80], it will be interesting to know if they will better support tumour niche and prolong viability of PDEs. Most PDEs were maintained at a temperature of 37°C and a CO_2_ level of 5%, which are similar to physiological parameters in the human body. There were variations in O_2_ level, with a number of studies opting for a hyperoxic environment presumably due to the fact that hypoxia reduces tissue viability, although it is difficult to measure the amount of O_2_ that will diffuse into the tissue in this experimental setup [78, 81].

With the almost endless combinations of culture methods and conditions, it is not surprising that the most optimal system for CRC explant cultures has not been established. In addition, different assays and quality standards were used to characterise tissue viability at various time points. Most studies reported on tissue morphology based on H&E stain, although careful quantification was sometimes lacking. In early studies, cell proliferation was assessed using autoradiography, which measured the ability to incorporate uridine and thymidine during DNA synthesis [49, 70]. Subsequently, this was replaced by immunohistochemical staining of proliferation markers such as Ki-67 [44, 46, 53, 55], which is a nuclear protein expressed in all phases of the cell cycle, except the resting phase (G0). More recently, newer techniques such as fluorescence-based viability assays [53, 54] and gene expression analysis [44, 56] have been utilised to further support the use of explants as a preclinical model. Regardless of culture methods and conditions, most studies demonstrated that CRC PDEs can be maintained ex vivo for at least 3–7 days without significant loss of viability. Remarkably, da Mata et al. was able to maintain their PDE cultures for a maximum of 122 days (median, 28 days), which may be partly explained by the use of a similar culture medium which has successfully established CRC organoids, as well as an agitation-based system which promotes diffusion of oxygen and soluble factors [54].

Given the main advantage of the explant model is the preservation of the TME, a better characterisation of this environment is crucial to understand the mechanisms for treatment response and provide new insight into potential resistance mechanisms. To this end, previous studies have used PDEs to investigate the roles of different enzymes [57, 58] and cytokines [15, 50, 59–63] which may contribute to cancer progression and metastasis. As expected, the cytokine profiles differed significantly between normal and tumour microenvironment. Consequently, the cytokine profile influences the immune cell populations present in the TME. Disappointingly, there has been a lack of studies that looked at immune cell subtypes and spatial relationships between tumour and immune cells [39, 50], despite these factors being predictive of chemotherapeutic drug responses and patient outcomes in CRC [9–11]. Recent results from pancreatic and ovarian PDEs have demonstrated that immune cells, such as macrophages, cytotoxic T lymphocytes and regulatory T cells, can be retained in cultures, ensuring that this platform will provide functional relevance and redefine evaluation of current and novel therapies [82–84].

Over the years, the use of explant cultures has predominantly focused on assessing ex vivo response to commonly used chemotherapeutic agents, and increasingly, targeted therapies and immunotherapies used in the clinical setting. Many studies have utilised the HDRA which can identify effective treatments based on inhibition of tumour viability [14, 16, 17, 19, 20, 23, 24, 27–30, 32, 33, 44, 65]. Briefly, it involves incubating tumour fragments on collagen or gelatin sponge, with or without drugs, for a specific time period and then adding MTT, which viable cells convert into purple formazan that is measured by spectrophotometry. Whilst this metabolic activity assay is easy to use and rapid and allows high throughput, the end results may be affected by different factors (e.g. pH, cellular ion concentration, cell types and numbers). In addition, MTT is insoluble in cell culture media and needs to be dissolved in dimethyl sulfoxide (DMSO) or isopropanol, thus it is mainly used as an endpoint detection method. IHC staining has also been commonly used to compare drug-treated and untreated PDEs. Treatment effects are quantified by calculating the percentage of cells expressing proliferation markers (e.g. Ki-67, PCNA) and apoptotic markers (e.g. cleaved-caspase-3), although the scoring may be subjective.

At present, only 7.5% of all potential anti-cancer drugs that enter phase I clinical trials are eventually approved for clinical use [85]. Undoubtedly, a major obstacle in new drug development and subsequent approval is the ability to predict clinical efficacy in preclinical models [86]. The potential ability to differentiate between treatment responders and non-responders can greatly assist in selection of patients for clinical trials. Potential biomarkers of treatment response, such as gene expression (TS, miRNA34, CHFR) and protein markers (e.g. p53, p21, ABCG2), were investigated but none of them have been validated in other studies. Moreover, many of them were identified from HDRA, instead of correlating with in vivo clinical response, so they must be interpreted with caution. A recent notable advance is the development of the CANScript platform by Majumder et al. which incorporated results from a combination of ex vivo assays (H&E; Ki-67 and cleaved caspase-3 on IHC; CCK-8 assay) to generate a score to predict clinical response with 91.67% specificity and 100% sensitivity [53].

As with any other preclinical models, the PDE model has its own challenges include obtaining sufficient tumour tissue, timely and careful processing of tissue, optimising culture method and conditions, and establishing the optimal window of time for drug testing or co-cultures. With rapid developments in biomolecular technologies (e.g. tumour-on-a-chip microfluidic platforms to screen multiple drugs on a single PDE) and endpoint analysis (e.g. multiplex immunofluorescence staining to differentiate cell types in the TME, non-destructive assays to permit readouts at multiple timepoints), it is hoped that some of these challenges can be overcome to bring back PDEs as a powerful preclinical model in cancer research.

At present, our scoping review on its utility in translational research remains inconclusive, largely because of the limited number and heterogeneity of previous studies. Further validation studies are required to characterise, refine and standardise this preclinical model before it can become a part of precision medicine. This will require close collaboration between clinicians and scientists to support ongoing research efforts.

## Conclusion

In summary, given the preservation of the TME and tumour heterogeneity, PDEs represent a more clinically relevant model for CRC than other preclinical models. In addition, they are relatively easy to establish, cost-effective, and provide results within a clinically acceptable timeframe. With the introduction of immunotherapies such as anti-PD1, which has shown excellent clinical efficacy in a subset of CRC patients, there has never been a stronger incentive to incorporate PDEs in future preclinical studies. We believe that the use of PDEs for functional assays, in combination with molecular profiling, has the potential to identify more reliable biomarkers, potential treatment resistance mechanisms and novel therapeutic agents to improve patient outcomes in CRC.

### Supplementary Information

Below is the link to the electronic supplementary material.

Supplementary file1 (DOCX 549 KB)

## Data Availability

All data supporting the findings of this study are available within the paper and its Supplementary Information.
