# Supplementary material for: Use of patient-derived explants as a preclinical model for precision medicine in colorectal cancer: A scoping review
Source: Langenbecks Arch Surg. 2023 Oct 10;408(1):392. doi: 10.1007/s00423-023-03133-7 (PMC10564805; doi:10.1007/s00423-023-03133-7)
Supplement: Supplementary file 1 — Supplementary file1 (DOCX 549 KB) [file 423_2023_3133_MOESM1_ESM.doc]

## **Supplementary Information**

**Literature search strategy**

| **Database** | **Search Strategy** | **Results** |
| --- | --- | --- |
| Ovid MEDLINE  (1946 to September 2022) | (colorectal cance* or colorectal neoplas* or colorectal tumo* or colorectal carcinom*).mp. [mp=title, abstract, original title, name of substance word, subject heading word, floating sub-heading word, keyword heading word, organism supplementary concept word, protocol supplementary concept word, rare disease supplementary concept word, unique identifier, synonyms]  AND  (explant or organotypic or slice or histoculture).mp. [mp=title, abstract, original title, name of substance word, subject heading word, floating sub-heading word, keyword heading word, organism supplementary concept word, protocol supplementary concept word, rare disease supplementary concept word, unique identifier, synonyms] | 274 |
| EMBASE  (1974 to September 2022) | (colorectal cance* or colorectal neoplas* or colorectal tumo* or colorectal carcinom*).mp. [mp=title, abstract, original title, name of substance word, subject heading word, floating sub-heading word, keyword heading word, organism supplementary concept word, protocol supplementary concept word, rare disease supplementary concept word, unique identifier, synonyms]  AND  (explant or organotypic or slice or histoculture).mp. [mp=title, abstract, original title, name of substance word, subject heading word, floating sub-heading word, keyword heading word, organism supplementary concept word, protocol supplementary concept word, rare disease supplementary concept word, unique identifier, synonyms] | 639 |
| Web of Science  (1900 to September 2022) | colorectal cance* or colorectal neoplas* or colorectal tumo* or colorectal carcinom*  AND  explant or organotypic or slice or histoculture | 691 |
| Cochrane Library  (Up to September 2022) | colorectal cance* or colorectal neoplas* or colorectal tumo* or colorectal carcinom*  AND  explant or organotypic or slice or histoculture | 37 |

**Supplementary Table 1.** CRC PDE culture techniques (n=60).

| **Author (year)** | **Sample size** | **Tissue source** | **Culture method** | **Culture medium/additives** | **Culture conditions** | **Culture duration (max.)** |
| --- | --- | --- | --- | --- | --- | --- |
| Rovin (1962) Error: Reference source not found | NR | Surgical specimen | Tumour fragments on either rayon acetate, stainless steel mesh or plasma clot | CMRL-1066, Eagle's medium+20% human serum, or chicken plasma+chicken embryo extract | 35-37°C; 0.3-0.4% CO2/20% O2, 5% CO2/1% O2, or 5% CO2/95% O2 | 8 days |
| Wolberg (1962) Error: Reference source not found | 11 | Surgical specimen | Tumour slices | Eagle's medium, 10% dialyzed bovine serum, penicillin, streptomycin | 37°C; 5% CO2/95% O2; agitation | 24 hours |
| Wright (1962) Error: Reference source not found | 6 | Surgical specimen | Tumour fragments in rooster plasma clots on coverslips | Eagle's medium, 20% autologous serum/non-dialysed pooled human serum, antibiotics | 37°C; agitation (12rph) | NR |
| Wolberg (1964) Error: Reference source not found | 11 | Surgical specimen | Tumour slices | Eagle's medium, 10% dialysed bovine serum, penicillin, streptomycin | 37°C; 5% CO2/95% air; agitation | 24 hours |
| Hurley (1965) Error: Reference source not found | 50 | Surgical specimen | Tumour fragments in chicken plasma clot on coverslips | Parker's TC-199, 20% FCS, antibiotics | 37°C; agitation (12rph) | NR |
| Roller (1966) Error: Reference source not found | 32 | Surgical specimen | Tumour fragments on Millipore filter strips placed on wire mesh grids | Eagle's MEM, 10% patient's serum, penicillin, streptomycin, Fungizone, L-glutamine | 37°C; 10% CO2/90% O2; 90% humidity | 11 days |
| Kalus (1972) Error: Reference source not found | 26 | Surgical specimen | Tumour fragments on human fibrin foam | 90% Eagle's based medium, 10% FCS, 1% penicillin/streptomycin | 37°C; 5% CO2/95% air; 98% humidity | 21 days |
| Pritchett (1982) Error: Reference source not found | 10 | Surgical specimen | Tumour fragments on stainless steel grids | Waymouth MB752/1 medium, 10-15% FCS, ascorbic acid, hydrocortisone-21-sodium succinate, ferrous sulphate, penicillin, streptomycin, mycostatin | 38°C; 5% CO2/95% O2 | 24 hours |
| Harvey (1988) Error: Reference source not found | 26 | Surgical specimen | Tumour fragments on plastic meshwork raft | RPMI 1640 | 37°C; 5% CO2/95% air | 2 days |
| Yamagata (1991) Error: Reference source not found | 15 | Surgical specimen | Tumour slices | RPMI 1640 | 37°C | 4 hours |
| Furukawa (1992) Error: Reference source not found | 36 | Surgical specimen | Tumour fragments on collagen sponge gels | RPMI 1640, 20% FCS, penicillin, gentamicin | 37°C; 5% CO2/95% air | 9 days |
| Furukawa (1995) Error: Reference source not found | 109 | Surgical specimen | Tumour fragments on collagen sponge gels | RPMI 1640, 20% FCS | 37°C; 5% CO2/95% air | 7 days |
| Hood (1998) Error: Reference source not found | 5 | Surgical specimen | Tumour slices | RPMI 1640, horse serum, FCS, penicillin, streptomycin, glutamine, amphotericin B, insulin-transferrin-selenium | 37°C; 5% CO2/95% air | 7 days |
| Takeda (1999) Error: Reference source not found | 22 | Surgical specimen | Tumour fragments on collagen sponge gels | RPMI 1640, 20% FCS, penicillin, streptomycin, amphotericin B | 37°C; 5% CO2/95% air | 7 days |
| Kanamori (1999) Error: Reference source not found | 19 | Endoscopic biopsies | Tumour fragments on collagen sponge gels | RPMI 1640, 20% FCS | 37°C; 5% CO2/95% air | 2 days |
| Hosaka (2001) Error: Reference source not found | 28 | Surgical specimen | Tumour fragments on collagen surface | NR | 37°C | 7 days |
| Isshi (2002) Error: Reference source not found | 62 | Surgical specimen | Tumour fragments on collagen sponge gels | RPMI 1640, 20% FCS | 37°C; 5% CO2 | 7 days |
| Yanqun (2002) Error: Reference source not found | 20 | Surgical specimen | Tumour fragments on collagen sponge gels | DMEM/MEM, 9% FBS, non-essential amino acids, gentamicin, cefotaxime | 37°C; 5% CO2/95% air | 6 days |
| Golby (2002) Error: Reference source not found | 24 | Surgical specimen | Tumour fragments on steel grids | 60% Trowell T8, 20% NCTC, 15% FCS, 2% L-glutamine, penicillin, streptomycin, HEPES | 37°C; 95% O2 | 3 days |
| Inoue (2005) Error: Reference source not found | 23 | Surgical specimen | Tumour fragments on gelatin sponges | RPMI 1640, 20% FCS | 37°C; 5% CO2 | 7 days |
| Matsuoka (2006) Error: Reference source not found | 29 | Surgical specimen | Tumour fragments suspended in thermoreversible gelation polymer | RPMI 1640, 10% FCS, penicillin, streptomycin, L-glutamine, amphotericin B, insulin | 37°C; 5% CO2 | 4 days |
| Kwon (2006) Error: Reference source not found | 35 | Surgical specimen or biopsies | Tumour fragments on collagen sponge gels | RPMI 1640, 20% FCS | 37°C; 5% CO2/95% air | 3 days |
| Monteleone (2006) Error: Reference source not found | 5 | Surgical specimen | Tumour fragments on steel grids | Serum-free media | 37°C; 5% CO2/95% O2 | 6 hours |
| Kwon (2007) Error: Reference source not found | 12 | Surgical specimen or biopsies | Tumour fragments on collagen sponge gels | RPMI 1640, 20% FCS | 37°C; 5% CO2/95% air | 3 days |
| Kinoshita (2007) Error: Reference source not found | 49 | Surgical specimen | Tumour fragments on gelatin sponges | RPMI 1640, 20% FCS | 37°C; 5% CO2 | 7 days |
| Stolfi (2008) Error: Reference source not found | 5 | Surgical specimen | Tumour fragments on steel grids | Serum-free media | 37°C; 5% CO2/95% O2 | 18 hours |
| Pirnia (2009) Error: Reference source not found | 6 | Surgical specimen | Tumour fragments on gelatin sponges | RPMI 1640, 10% FBS, antibiotic, antimycotic | 37°C; 5% CO2 | 5 days |
| Kim (2009) Error: Reference source not found | 114 | Surgical specimen | Tumour fragments on gelatin sponges | RPMI 1640, 20% FCS | 37°C; 5% CO2 | 3 days |
| Yuan (2009) Error: Reference source not found | 22 | Surgical specimen | Tumour fragments on filter paper | RPMI 1640 | 37°C; 5% CO2 | 5 days |
| Vaira (2010) Error: Reference source not found | 49 | Surgical specimen | Tumour slices on Millipore inserts | Ham’s F-12, 20% FBS, penicillin, streptomycin, amphotericin B, kanamycin | 37°C; 5% CO2 | 5 days |
| Brouquet (2011) Error: Reference source not found | 64 | Surgical specimen | Tumour fragments on polyHEMA | Complete medium | NR | 7 days |
| Michielsen (2011) Error: Reference source not found | 21 | Surgical specimen | Tumour fragments | RPMI 1640, 20% FCS, penicillin, streptomycin, gentamicin, amphotericin B | NR | 3 days |
| Lee (2012) Error: Reference source not found | 76 | Surgical specimen | Tumour fragments on collagen sponge gels | RPMI 1640, 20% FCS, amphotericin B | 37°C; 5% CO2 | 7 days |
| Yoon (2012) Error: Reference source not found | 324 | Surgical specimen | Tumour fragments on gelatin sponges | RPMI 1640, 20% FCS | 37°C; 5% CO2 | 3 days |
| Stolfi (2012) Error: Reference source not found | 6 | Surgical specimen | Tumour fragments on steel grids | RPMI 1640, 10% FBS, penicillin, streptomycin, gentamicin | 37°C; 5% CO2/95% O2 | 22 hours |
| Muthuswamy (2012) Error: Reference source not found | 72 | Surgical specimen | Tumour fragments | IMDM, 10% FBS, gentamicin | 37°C; 5% CO2 | 2 days |
| Zhang (2014) Error: Reference source not found | 50 | Surgical specimen | Tumour fragments on collagen sponge gels | RPMI 1640, 20% FCS | 37°C; 5% CO2 | 7 days |
| Stolfi (2014) Error: Reference source not found | 5 | Surgical specimen | Tumour fragments on Millicell inserts | RPMI 1640, 10% FBS, penicillin, streptomycin, gentamicin | 37°C; 5% CO2/95% O2 | 1 day |
| O'Toole (2014) Error: Reference source not found | 50 | Surgical specimen | Tumour fragments | RPMI 1640, 20% FCS, penicillin, streptomycin, gentamicin, amphotericin B | NR | 3 days |
| Majumder (2015) Error: Reference source not found | 52 | Surgical specimen or biopsies | Tumour slices on tumour-stromal matrix proteins | RPMI, 2% autologous serum, 8% FBS, penicillin, insulin-transferrin-selenium, GlutaMAX™, streptomycin, amphotericin B | NR | 3 days |
| Unger (2015) Error: Reference source not found | 4 | Surgical specimen | Tumour slices | RPMI 1640, L-glutamine, sodium selenite, ethanolamine, phosphorylethanolamine, triiodothyronine, sodium pyruvate, MEM-vitamins, penicillin, streptomycin, gentamicin, amphotericin B, transferrin, bovine serum albumin, EGF, fetuin, insulin, hydrocortisone, FCS | 37°C; 5% CO2; agitation (100rpm) | 3 days |
| Murphy (2016) | 40 | Surgical specimen | Tumour fragments | RPMI, 20% FCS, penicillin, streptomycin, gentamicin, amphotericin B | 37°C; 5% CO2 | 4 days |
| Flanagan (2016) Error: Reference source not found | 19 | Surgical specimen | Tumour fragments on gelatin sponges | DMEM, penicillin, streptomycin, amphotericin B, ciprofloxacin and gentamicin | 37°C; 5% CO2 | 5 days |
| Yoon (2017) Error: Reference source not found | 129 | Surgical specimen | Tumour fragments on gelatin sponges | RPMI-1640, 10% FBS | 37°C; 5% CO2/95% air | 5 days |
| Ji (2017) Error: Reference source not found | 89 | Surgical specimen | Tumour fragments on collagen sponge gels | RPMI 1640, 20% FBS, amphotericin B | 37°C; 5% CO2 | 7 days |
| Brijwani (2017) Error: Reference source not found | 40 | Surgical specimen or biopsies | Tumour slices | RPMI 1640, 20% FBS, insulin-transferrin-selenium, GlutaMAX™, penicillin, streptomycin, amphotericin B | NR | 3 days |
| Kistner (2017) Error: Reference source not found | 22 | Surgical specimen | NR | DMEM, 10% FCS, penicillin, streptomycin, L-glutamine | 37°C; 7% CO2 | 22 hours |
| Sonnichsen (2018) Error: Reference source not found | 7 | Surgical specimen | Tumour slices on membrane | RPMI 1640, 10% FCS, L-glutamine, penicillin, streptomycin, amphotericin B | 37°C; 5% CO2 | 3 days |
| Li (2018) Error: Reference source not found | 19 | Surgical specimen | Tumour fragments on collagen sponge gels | RPMI 1640 | NR | 7 days |
| Lo Re (2018) Error: Reference source not found | NR | Surgical specimen | Tumour slices | Advanced DMEM/Ham’s F-12, HEPES, GlutaMAX™, B-27 without retinoic acid, N-2, NAC, EGF | 37°C; 5% CO2 | 3 days |
| Benkhelifa (2019) Error: Reference source not found | 34 | Surgical specimen | Tumour fragments | DMEM medium, 10% FBS, L-glutamine, penicillin, streptomycin | 37°C; 5% CO2 | 1 day |
| Butler (2019) | 15 | Surgical specimen | Tumour fragments | RPMI 1640, 10% FBS, penicillin, streptomycin, gentamicin, amphotericin B | 37°C; 5% CO2 | 3 days |
| Laudisi (2019) Error: Reference source not found | 26 | Surgical specimen | Tumour fragments on Millicell inserts | RPMI 1640, 10% FBS, penicillin, streptomycin, gentamicin | 37°C; 5% CO2/95% O2 | 1 day |
| Ott (2019) Error: Reference source not found | 27 | Surgical specimen | Tumour fragments | RPMI/Ham’s F-12, bovine serum albumin, penicillin, streptomycin | 5% CO2/95% O2; agitation (low speed) | 1 day |
| Ahmed (2020) Error: Reference source not found | 7 | Surgical specimen | Tumour fragments | Advanced DMEM/Ham’s F-12, B-27, NAC, nicotinamide, EGF, A83-01, SB202190, Primocin, Noggin | NR | 2 days |
| Hewitt (2020) [48] | 3 | Surgical specimen | Tumour slices on Millicell inserts | RPMI 1640, 10% FBS, penicillin, streptomycin | NR | 5 days |
| Mutala (2021) Error: Reference source not found | 96 | NR | Tumour fragments | RPMI/Ham’s F-12, bovine serum albumin, penicillin, streptomycin | 5% CO2/95% O2; agitation (low speed) | 1 day |
| da Mata (2021) Error: Reference source not found | 26 | Surgical specimen | Tumour fragments | DMEM/Ham’s F-12, Primocin, B-27, gastrin, prostaglandin, nicotinamide, NAC, EGF | 37°C; 5% CO2; agitation (100rpm) | 122 days |
| Hagiwara (2022) Error: Reference source not found | 44 | Surgical specimen | Tumour fragments on gelatin sponges | RPMI 1640, 20% FCS | 37°C; 5% CO2/95% air | 7 days |
| Gavert (2022) Error: Reference source not found | 28 | Surgical specimen or biopsies | Tumour slices on titanium grids | DMEM/F12, 5% FCS, penicillin, streptomycin, amphotericin B, gentamicin, L-glutamine | 37°C; 5% CO2/80% O2; agitation (70rpm) | 5 days |

NR = not reported; rph =revolutions per hour; FBS/FCS = foetal bovine serum/foetal calf serum; MEM = Minimum Essential Medium; RPMI 1640 = Roswell Park Memorial Institute 1640; DMEM = Dulbecco's Modified Eagle Medium; HEPES = N-2-hydroxyethylpiperazine-N'-2-ethanesulphonic acid; IMDM = Iscove's Modified Dulbecco's Medium; EGF = epidermal growth factor; rpm = revolutions per minute; NAC = N-Acetyl Cysteine

**Supplementary Table 2.** Assessing *ex vivo* drug responses using CRC PDEs (n=38).

| **Author (year)** | **Drug (s)** | **Assay (s) performed** | **Outcome (s)** |
| --- | --- | --- | --- |
| Wolberg (1962) Error: Reference source not found | - 5-FU - FUDR - Azaserine - 6-thioguanine | - Autoradiography (for DNA synthesis) | - Changes in nucleoside incorporation produced by drugs varied greatly - Fluorinated pyrimidines inhibited uptake of uridine in most tumours but also increased uptake of thymidine in about two-thirds |
| Wolberg (1964) Error: Reference source not found | - 5-FU - FUDR | - Autoradiography (for DNA synthesis) | - In presence of fluorinated pyrimidines, nucleoside incorporation was either unaffected or decreased, but in case of thymidine, in many instances it was increased |
| Pritchett (1982) Error: Reference source not found | - Vincristine | - H&E | - Doses of 0.1, 0.5, 1.0 and 1.5ug/ml vincristine produced typical arrested metaphases in mucosa and tumour - Minimum dose for complete arrest in mucosa was taken to be 0.5ug/ml, while 3.0 ug/ml dose was the lowest at which no evidence of metaphase escape was apparent in tumours |
| Furukawa (1992) Error: Reference source not found | - MMC - Doxorubicin - 5-FU - Cisplatin | - HDRA | - Efficacy rates of MMC, doxorubicin, 5-FU and cisplatin were 20%, 5.7%, 20%, and 5.7% respectively - HDRA with MTT endpoint had higher evaluability and clinical correlativity compared to cell-suspension assay |
| Furukawa (1995) Error: Reference source not found | - MMC - Doxorubicin - 5-FU - Cisplatin | - HDRA | - Efficacy rates of MMC, doxorubicin, 5-FU and cisplatin were 17%, 7.5%, 24.5%, and 3.7% respectively |
| Kanamori (1999) Error: Reference source not found | - 5-FU | - HDRA | - 16.7% were sensitive to 5-FU |
| Hosaka (2001) Error: Reference source not found | - Cisplatin - 5-FU - MMC - Doxorubicin | - HDRA - TUNEL | - MMC was the most effective drug (40% efficacy rate), 5-FU was second most effective (37%), and cisplatin had poor efficacy (4%) - Apoptotic index of 5-FU and MMC was well correlated with inhibition rate, so their effects were related to apoptosis |
| Isshi (2002) Error: Reference source not found | - 5-FU | - HDRA | - 30% were sensitive to 5-FU |
| Yanqun (2002) Error: Reference source not found | - Sodium salicylate | - IHC (BrdU) | - 60% showed chemosensitivity within clinically relevant concentration range - Doubling exposure time decreased IC50, suggesting that a similar inhibition might be achieved with a lower concentration over an extended time - None of the right-sided cancers was sensitive |
| Matsuoka (2006) Error: Reference source not found | - 5-FU | - WST-8 assay | - 47.6% were sensitive to 5-FU; 52.4% had no sensitivity |
| Kwon (2006) Error: Reference source not found | - K101 (trans,cis-Pt(acetato)2Cl2(1,4-butanediamine)) - Cisplatin | - HDRA | - K101 showed 80.0-91.4% efficacy rate, compared with 48.6% for cisplatin |
| Monteleone (2006) Error: Reference source not found | - 5-ASA | - Western blot - PTP activity assay | - 5-ASA caused a two-fold increase in activity of p-EGFR-targeting phosphatases and reduced p-EGFR in CRC cells |
| Kwon (2007) Error: Reference source not found | - K104 (trans-[Pt(trifluoroacetato)2(malonate)(butanediamine)]) - Cisplatin - Carboplatin | - HDRA | - K104 showed a 50.0-66.7% efficacy rate, compared with 33.3% for cisplatin and 58.3% for carboplatin |
| Stolfi (2008) Error: Reference source not found | - Mesalazine | - Western blot | - Mesalazine significantly reduced CDC25A protein expression |
| Kim (2009) Error: Reference source not found | - 5-FU+leucovorin (FL) - Capecitabine - 5-FU+leucovorin+irinotecan (FLIRI) - 5-FU+leucovorin+oxaliplatin (FLOX) - HDAC inhibitors (suberoylanilide hydroxamic acid, PXD101, CG-1, CG-2, CG-3) | - HDRA | - Inhibition rate with established regimens was most significant for FLOX, followed by FL, FLIRI, 5-FU, and capecitabine - Inhibition rate with HDAC inhibitors was highest for PXD101, followed by SAHA, CG-1, CG-2, and CG-3 - Chemosensitivity with established regimens was between 34.2% and 52.6%, and between 54.5% and 84.1% with HDAC inhibitors - All HDAC inhibitors displayed synergistic effects in combination with established regimens of FLOX and FLIRI |
| Yuan (2009) Error: Reference source not found | - Epirubicin - Cisplatin - Oxaliplatin - 5-FU - Taxetere - Irinotecan - 5-FU+epirubicin+cisplatin - 5-FU+irinotecan - 5-FU+oxaliplatin - 5-FU+taxetere+cisplatin | - HDRA | - Among single agents, inhibition rate was highest for oxaliplatin (17.5%), and sensitivity was highest for 5-FU (36.4%) - In combination groups of agents, inhibition rate was highest for 5-FU+oxaliplatin (54.1%), and sensitivity was highest for 5-FU+epirubicin+cisplatin and 5-FU+taxetere+cisplatin (71.4%) - Inhibition rates and sensitivity to combined agents were higher than those of single agents |
| Vaira (2010) Error: Reference source not found | - LY294002 (PI3K inhibitor) | - IHC (Ki-67, Akt and S6RP) - TUNEL - MTT assay - RT-PCR (PI3K, AKT1, S6RP), gene expression analysis | - Treated tumour slices showed a remarkable reduction of p-Akt and p-S6RP levels at all time points and reduction in proliferation rate and tumour cell viability without affecting gene expression of the same targets |
| Brouquet (2011) Error: Reference source not found | - SN-38 (topoisomerase I inhibitor metabolite) | - IHC (Ki-67, AE1/AE3) | - Culture with SN-38 was associated with a significant decrease in proliferative index (22% vs. 51%) - 34% were highly sensitive to SN-38 (DRI >70%); 18% were not sensitive (DRI 30% or less) |
| Um (2012) Error: Reference source not found | - 5-FU - Irinotecan - Oxaliplatin | - HDRA | - Inhibition rates for 5-FU, irinotecan and oxaliplatin were 55.6%, 34.8% and 45.3%, respectively |
| Yoon (2012) Error: Reference source not found | - 5-FU+leucovorin - 5-FU+leucovorin+oxaliplatin (FOLFOX) - 5-FU+leucovorin+irinotecan (FOLFIRI) - 5-FU+leucovorin+oxaliplatin+ bevacizumab - 5-FU+leucovorin+oxaliplatin+ cetuximab - 5-FU+leucovorin+irinotecan+ bevacizumab - FU+leucovorin+irinotecan+ cetuximab | - HDRA | - Inhibition rates of regimes using FOLFOX (34.2-39.2%) were higher than those using FOLFIRI |
| Stolfi (2012) Error: Reference source not found | - 2-14 (2-methoxy-5-amino-N-hydroxybenzamide) | - ELISA - PCR - Western blot | - 2-14 significantly reduced COX-2 expression, both at RNA and protein level, and PGE2 protein expression |
| Zhang (2014) Error: Reference source not found | - Raltitrexed - Pemetrexed - 5-FU | - HDRA | - Mean inhibition rates for raltitrexed, pemetrexed and 5-FU were 37.56%, 41.53% and 42.82%, respectively |
| Stolfi (2014) Error: Reference source not found | - Smad7 antisense oligonucleotide | - IHC (Smad7, PCNA, p-eIF2α, CDC25A) | - Inhibition of Smad7 with Smad7 antisense oligonucleotide reduced fraction of Smad7-, PCNA- and CDC25A-expressing CRC cells and increased percentage of cells expressing p-eIF2α |
| Majumder (2015) Error: Reference source not found | - Cetuximab | - H&E - IHC (Ki-67 and cleaved caspase-3) - CCK-8 assay | - Cetuximab-treated group showed a decrease in cell viability and Ki-67 positivity |
| Unger (2015) Error: Reference source not found | - Staurosporine - Gefitinib | - Cell viability assay (ATP) - Cell apoptosis assay (caspase 3/7) - Multiplex protein arrays - IHC (EGFR) - Western blot | - Staurosporine and gefitinib showed a dose-dependent reduction of viability and downstream signalling pathways (Akt and MAPK kinase phosphorylation) |
| Murphy (2016) | - Quininib | - ELISA | - Quininib significantly reduced secretions of angiogenic mediators (IL-6, IL-8 and VEGF), as well as other mediators (ENA-78, GRO-a, TNF, IL-1b and MCP-1) |
| Flanagan (2016) Error: Reference source not found | - 5-FU+oxaliplatin - 5-FU+oxaliplatin+Ac-DLND-CHO (caspase-3 inhibitor) - 5-FU+oxaliplatin+aspirin - 5-FU+oxaliplatin+ celecoxib | - IHC (caspase-3, β-catenin, Ki-67) - IF (active caspase-3, Ki-67) - Apoptosis assay - Enzyme immunoassay (PGE2) | - 5-FU-based chemotherapy increased percentage of cells positive for active caspase-3 and TUNEL, but also expression of regeneration and proliferation markers β-catenin and Ki-67, as well as COX-2 - Inhibition of caspase-3 and COX-2 resulted in a reduction in expression of proliferation markers - 5-FU-based regimen caused a significant increase in PGE2 release, while specific Caspase-3 and COX-2 inhibition reduced PGE2 levels |
| Yoon (2017) Error: Reference source not found | - 5-FU+leucovorin+oxaliplatin (FX) - 5-FU+leucovorin+irinotecan (FR) - 5-FU+leucovorin+oxaliplatin+ bevacizumab - 5-FU+leucovorin+oxaliplatin+ cetuximab - 5-FU+leucovorin+irinotecan+ bevacizumab - 5-FU+leucovorin+irinotecan+ cetuximab | - Integrative tumour response assay (sequential HDRAs) | - Inhibition rates with first-line regimens for FR and FX were 36% and 32.6%, respectively - Inhibition rates for second-line regimens were lower than those of first-line regimens - Inhibition rates for first-line FR was significantly better than that for FX - Differences in inhibition rates between first- and second-line regimens were significant for FR, but not for FX - For second-line treatment, switching from FX to FR or from FR to FX and adding a targeted agent resulted in better inhibition rates than adding a targeted drug to first-line regimen - In terms of targeted agents, adding cetuximab to second-line regimens resulted in significantly better inhibition rate than adding bevacizumab |
| Ji (2017) Error: Reference source not found | - 5-FU | - HDRA | - 57.3% were sensitive to 5-FU |
| Brijwani (2017) Error: Reference source not found | - Cetuximab - Trastuzumab - MK0752 (Notch inhibitor) - Cetuximab+trastuzumab - Cetuximab+MK0752 - Trastuzumab+MK0752 | - H&E - IHC (Ki-67, caspase-3) - CCK-8 assay | - Tumours that were segregated as responders to cetuximab (22%) showed a significant reduction in viability/tumour content or proliferation and a concomitant increase in activated caspase-3 post-treatment - In contrast, most tumours (78%), when treated with cetuximab, did not exhibit significant changes in viability, proliferation and apoptosis; they were categorized as non-responders - 76% responded to dual combination of trastuzumab and MK0752 |
| Sonnichsen (2018) Error: Reference source not found | - 5-FU - 5-FU+oxaliplatin | - H&E - IF (AE1/AE3, Ki-67) | - Tissue slices treated with 5-FU showed lower tumour cell fractions and dose-dependent alterations of proliferating tumour cells, compared with controls - Individual tumour samples were examined and differences in chemotherapy susceptibility could be observed |
| Lo Re (2018) Error: Reference source not found | - Traniplatin - Cisplatin | - IHC (caspase-3, CD45) | - Traniplatin-treated explants had increased apoptosis and presence of CD45+ cells, suggesting decreased cytotoxic activity against immune cells - Only a few isolated CD45+ cells were observed in most cisplatin-treated explants |
| Butler (2019) | - Q8 (1,4 dihydroxy quininib) | - ELISA | - Q8 significantly decreased secretions of soluble VCAM-1 and TIE-2 proteins |
| Laudisi (2019) Error: Reference source not found | - Progranulin antisense oligonucleotide | - IHC (p-STAT3, Ki-67, progranulin) | - Progranulin inhibition reduced fraction of transformed cells expressing Ki67, as well as p-STAT3 Tyr705-expressing cells |
| Ahmed (2020) Error: Reference source not found | - AM404 (anandamide uptake inhibitor) | - H&E - IHC (Ki-67, caspase-3, CDX2, CD44) | - AM404 significantly reduced proliferation and stemness levels, and increased differentiation level in tumour explants |
| Hewitt (2020) [48] | - MEDI1191 (human IL12 mRNA) | - IHC (CD3, NKp46) - ELISA - MesoScale Discovery assay - Gene expression analysis | - MEDI1191 induced dose-dependent IL-12 production, IFN-γ production, and Th1 gene expression |
| Da Mata (2021) Error: Reference source not found | - 5-FU - 5-FU+oxaliplatin | - H&E - LDH cytotoxicity assay | - Tumour and stroma cell viability was maintained in control, whereas no viable tumour cells were detected and viable stromal cells were sometimes present in those exposed to 5-FU - There was increased drug-induced cell death in PDEs challenged with 5-FU, compared to control - Differences were also observed for PDEs challenged with a combination of 5-FU and oxaliplatin |
| Gavert (2022) Error: Reference source not found | - 5-FU+irinotecan (FOLFIRI) - 5-FU+oxaliplatin (FOLFOX) - Selumetinib - Bosutinib - Selumetinib+bosutinib - Selumetinib+bosutinib+5-FU+irinotecan - Selumetinib+bosutinib+5-FU+oxaliplatin | - H&E | - Each tumour responded differently to different drugs and different tumours responded very differently to same drug - Combination of MEK inhibitor selumetinib and Src inhibitor bosutinib, especially when combined with FOLFOX or FOLFIRI, was highly effective in a subset of tumours |

5-FU = fluorouracil; FUDR = 5-fluoro-2-deoxyuridine; H&E = haematoxylin and eosin; MMC = mitomycin C; HDRA = histoculture drug response assay; MTT = 3-(4,5-dimethylthiazol-2-yl)-2,5-diphenyltetrazolium bromide; TUNEL = terminal deoxynucleotidyl transferase dUTP nick end labelling; IHC = immunohistochemistry; BrdU = bromodeoxyuridine; IC50 = half-maximal inhibitory concentration; WST-8 = water-soluble tetrazolium 8; 5-ASA = 5-aminosalicylic acid; PTP = protein tyrosine phosphatase; p-EGFR = phosphorylated epidermal growth factor receptor; CDC25A = cell division cycle 25 A; HDAC = histone deacetylase; PI3K = phosphoinositide 3-kinase; Akt = protein kinase B; S6RP = S6 ribosomal protein; RT-PCR = reverse transcription-polymerase chain reaction; DRI = dose reduction index; ELISA = enzyme-linked immunosorbent assay; COX-2 = cyclooxygenase-2; PGE2 = prostaglandin E2; PCNA = proliferating cell nuclear antigen; p-eIF2a = phospho-eukaryotic initiation factor 2a; CCK-8 = cell counting kit-8; ATP = adenosine triphosphate; MAPK = mitogen-activated protein kinase; VEGF = vascular endothelial growth factor; ENA78 = epithelial neutrophil activating peptide 78; GRO-a = growth-regulated oncogene alpha; TNF = tumour necrosis factor; MCP-1 = monocyte chemoattractant protein-1; IF = immunofluorescence; VCAM-1 = vascular cell adhesion molecule-1; TIE-2 = TEK receptor tyrosine kinase; p-STAT3 = phosphorylated signal transducer and activator of transcription 3; IFN-γ = interferon-gamma; LDH = lactate dehydrogenase; MEK = mitogen-activated extracellular signal-regulated kinase

**Supplementary Table 3.** Biomarker discovery and validation using CRC PDEs (n=24)

| **Author (year)** | **Intervention** | **Biomarker (s)** | **Outcome measure** | **Results** |
| --- | --- | --- | --- | --- |
| Wolberg (1962) Error: Reference source not found | - 5-FU - FUDR - Azaserine - 6-thioguanine | - Uridine and thymidine uptake (on autoradiography) | - Treatment response (based on clinical response) | - Patients who did not respond to chemotherapy had tumours which showed either poor nucleoside incorporation, poor inhibition of uridine incorporation produced by drugs, or stimulation of thymidine incorporation even though drugs inhibited uridine uptake |
| Wright (1962) Error: Reference source not found | - Methotrexate - 5-FU - TSPA - Chlorambucil - Phenylalanine nitrogen mustard - Dihydro E-73 - Actinomycin D - Aminonucleoside of puromycin | - Cellular changes (on Jenner-Giemsa stain) | - Treatment response (based on clinical response) | - Clinical correlation with tissue culture was positive in 50.4%, equivocal in 22.1%, and negative in 27.5% - Methotrexate, TSPA, and dihydro E-73 showed a higher degree of correlation than chlorambucil, actinomycin D, 5-FU, or aminonucleoside of puromycin |
| Wolberg (1964) Error: Reference source not found | - 5-FU - FUDR | - Uridine and thymidine uptake (on autoradiography) | - Treatment response (based on clinical response) | - None of the tumours which incorporated neither uridine nor thymidine into DNA responded to systemic fluorinated pyrimidine chemotherapy |
| Hurley (1965) Error: Reference source not found | - Nitrogen mustard - Chlorambucil - Thio-Tepa - Methotrexate - 5-FU - Actinomycin D - Mithramycin - Streptonigrin - Velban | - Morphological criteria of damage (on H&E) | - Treatment response (based on clinical response) | - Clinical correlation with tissue culture was positive in 65% |
| Furukawa (1995) Error: Reference source not found | - MMC - Doxorubicin - 5-FU - Cisplatin | - Inhibition rate (using HDRA with MTT endpoint) | - Treatment response (based on clinical response) - Cancer recurrence/survival | - Correlation rate of HDRA to clinical drug sensitivity response was 92.1%, with 100% sensitivity and 90.6% specificity^#^ - Survival and recurrence-free survival rates were better in patients sensitive to 5-FU than those insensitive to 5-FU *in vitro* |
| Takeda (1999) Error: Reference source not found | - 5-FU - MMC - Cisplatin | - Serum p53 antibody | - Treatment response (based on HRDA) | - Inhibition index of 5-FU and cisplatin in seropositive group was significantly lower than that in seronegative group - Significant statistical differences in chemosensitivity to 5-fluorouracil and cisplatin were revealed depending on presence of serum p53 antibodies |
| Kanamori (1999) Error: Reference source not found | - 5-FU | - Inhibition rate (using HDRA with MTT endpoint) | - Treatment response (based on H&E from surgical specimens) | - True-positive rate and true-negative rate of HDRA were 33.3% and 88.9%, respectively, giving a high concordance rate (75%) between *in vitro* and *in vivo* chemosensitivity tests |
| Hosaka (2001) Error: Reference source not found | - Cisplatin - 5-FU - MMC - Doxorubicin | - p53 & p21 (on IHC) | - Treatment response (based on HRDA) | - With 5-FU and MMC, p53 labelling index was inversely correlated with inhibition rate (i.e. cases expressing normal p53 are chemosensitive to MMC and 5-FU) - With 5-FU and MMC, p21 labelling index showed good correlation with inhibition rate (i.e. cases expressing normal p21 are chemosensitive to MMC and 5-FU) |
| Isshi (2002) Error: Reference source not found | - 5-FU | - DPD & OPRT activity (on radioassay) | - Treatment response (based on HRDA) | - Mean OPRT enzyme activity was significantly higher in specimens testing positive by HDRA than in those specimens testing negative - Combination of DPD and OPRT levels may be predictive of 5-FU positive sensitivity |
| Inoue (2005) Error: Reference source not found | - 5-FU | - TS gene expression (on RT-PCR) | - Treatment response (based on HRDA) | - Significant increase in TS expression score was observed in 5-FU-sensitive CRC compared to 5-FU-resistant ones |
| Matsuoka (2006) Error: Reference source not found | - 5-FU | - WST-8 assay | - Treatment response (based on clinical response) - Median & cumulative survival | - Sensitivity of 5-FU testing was 90%, specificity was 90.9%, and diagnostic accuracy rate was 90.4% - Median survival times were longer in 5-FU sensitive group, compared to 5-FU insensitive and no chemotherapy groups - Cumulative survival rate was significantly higher in 5-FU sensitive group compared to 5-FU insensitive group |
| Kinoshita (2007) Error: Reference source not found | - 5-FU | - DPD & OPRT gene expressions (on RT-PCR) | - Treatment response (based on HRDA) | - mRNA expression of DPD had a weak tendency to be lower among responders - Difference in OPRT/DPD ratio had a weak tendency to be higher for responders |
| Kim (2009) Error: Reference source not found | - 5-FU+leucovorin (FL) - Capecitabine - 5-FU+leucovorin+irinotecan (FLIRI) - 5-FU+leucovorin+oxaliplatin (FLOX) - HDAC inhibitors (suberoylanilide hydroxamic acid, PXD101, CG-1, CG-2, CG-3) | - MMR defects | - Treatment response (based on HRDA) | - Tumours with MMR defects were closely correlated with chemosensitivities to combined regimens of PDX101 with FLOX and FLIRI |
| Yuan (2009) Error: Reference source not found | - Epirubicin - Cisplatin - Oxaliplatin - 5-FU - Taxetere - Irinotecan - 5-FU+epirubicin+cisplatin - 5-FU+irinotecan - 5-FU+oxaliplatin - 5-FU+taxetere+cisplatin | - ABCG2 (on IHC) | - Treatment response (based on HRDA) | - Expression of ABCG2 protein was in negative correlation to drug resistance to epirubicin |
| Brouquet (2011) Error: Reference source not found | - SN-38 (irinotecan-active metabolite) | - Ki-67 & AE1/AE3 (on IHC) | - Treatment response (based on clinical response using RECIST criteria) | - There was a non-significant trend towards correlation between *in vitro* response to SN-38 and *in vivo* response of colorectal liver metastases to CPT-11 (irinotecan) based chemotherapy |
| Yoon (2012) Error: Reference source not found | - 5-FU+leucovorin - 5-FU+leucovorin+oxaliplatin (FOLFOX) - 5-FU+leucovorin+irinotecan (FOLFIRI) - 5-FU+leucovorin+oxaliplatin+ bevacizumab - 5-FU+leucovorin+oxaliplatin+ cetuximab - 5-FU+leucovorin+irinotecan+ bevacizumab - FU+leucovorin+irinotecan+ cetuximab | - Inhibition rate (using HDRA with MTT endpoint) | - Treatment response (based on clinical response using RECIST criteria) | - Correlation rate of HDRA to clinical effect of chemotherapy was 66.3%, with 72.7% sensitivity and 54.7% specificity |
| Zhang (2014) Error: Reference source not found | - Raltitrexed - Pemetrexed - 5-FU | - TS gene expression (on RT-PCR) | - Treatment response (based on HRDA) | - TS mRNA levels were lower in pemetrexed/raltitrexed/5-FU-sensitive groups than resistant groups |
| Majumder (2015) Error: Reference source not found | - Cetuximab - Cetuximab+5-FU+leucovorin irinotecan | - S-score (H&E, Ki-67 and cleaved caspase-3 on IHC & CCK-8 assay) | - Treatment response (based on clinical response using PERCIST guidelines) | - CANScript-based model predicted clinical response with 91.67% specificity and 100% sensitivity* |
| Yoon (2017) Error: Reference source not found | - 5-FU+leucovorin+oxaliplatin (FX) - 5-FU+leucovorin+irinotecan (FR) - 5-FU+leucovorin+oxaliplatin+ bevacizumab - 5-FU+leucovorin+oxaliplatin+ cetuximab - 5-FU+leucovorin+irinotecan+ bevacizumab - 5-FU+leucovorin+irinotecan+ cetuximab | - Inhibition rate (using HDRA with MTT endpoint) | - Treatment response (based on clinical response using RECIST criteria) | - Correlation of HRDAs of first-line chemotherapeutic regimens with clinical response was 70.9%, with a sensitivity of 71.4% and specificity of 70.3% - Accuracy of ITRA for predicting response to second-line chemotherapy was 61.9%, with a sensitivity of 44.4% and specificity of 75% |
| Ji (2017) Error: Reference source not found | - 5-FU | - Inhibition rate (using HDRA with MTT endpoint) | - Disease-free survival | - There was a significant difference between 5-year DFS rates of chemo-sensitive and chemo-resistant groups |
| Brijwani (2017) Error: Reference source not found | - Cetuximab - Trastuzumab - MK0752 (Notch inhibitor) - Cetuximab+trastuzumab - Cetuximab+MK0752 - Trastuzumab+MK0752 | - AREG & EREG gene expressions (on RT-PCR) | - Treatment response (based on S-score) | - There was a strong link between low levels of EGFR ligands (AREG and EREG) expression in 22% of non-responders and cetuximab insensitivity |
| Li (2018) Error: Reference source not found | - Cisplatin - SN-38 (irinotecan-active metabolite) | - miR-34a gene expression (on RT-PCR) | - Treatment response (based on HDRA) | - Lower miR-34a expression was more resistant to cisplatin- or SN-38-based chemotherapy |
| Hagiwara (2022) Error: Reference source not found | - 5-FU - SN-38 (irinotecan-active metabolite) | - CHFR-promoter methylation (on PCR) | - Treatment response (based on HDRA) | - CHFR-promoter methylation significantly-positively correlated with inhibition of colon cancer by SN38 |
| Gavert (2022) Error: Reference source not found | - 5-FU+irinotecan (FOLFIRI) - 5-FU+oxaliplatin (FOLFOX) - Selumetinib - Bosutinib - Selumetinib+bosutinib - Selumetinib+bosutinib+5-FU+irinotecan | - H&E - Phosphorylated Src (on IHC) | - Treatment response (based on PDX model & clinical response) - Treatment response to MEK and Src inhibition (based on H&E) | - *Ex vivo* culture could accurately predict *in vivo* results of PDX models^+^ - *Ex vivo* culture response to chemotherapy correlated with clinical course in selected patients - Pre-treatment phosphorylated Src was identified as a predictive biomarker for MEK and Src inhibition (in absence of KRAS G12 mutation) |

5-FU = fluorouracil; FUDR = 5-fluoro-2-deoxyuridine; TSPA = triethylenethiophosphoramide; H&E = haematoxylin and eosin; MMC = mitomycin C; HDRA = histoculture drug response assay; MTT = 3-(4,5-dimethylthiazol-2-yl)-2,5-diphenyltetrazolium bromide; IHC = immunohistochemistry; DPD = dihydropyrimidine dehydrogenase; OPRT = orotate phosphoribosyltransferase; TS = thymidylate synthase; RT-PCR = reverse transcription-polymerase chain reaction; WST-8 = water-soluble tetrazolium 8; MMR = mismatch repair; ABCG2 = ATP binding cassette subfamily G member 2; RECIST = response evaluation criteria in solid tumours; CCK-8 = cell counting kit-8; PERCIST = PET response criteria in solid tumours; ITRA = integrative tumour response assay; AREG = amphiregulin; EREG = epiregulin; EGFR = epidermal growth factor receptor; miR-34a = microRNA 34a; CHFR = checkpoint-with-forkhead-and-ring-finger-domains; Src = proto-oncogene tyrosine-protein kinase SRC; MEK = mitogen-activated extracellular signal-regulated kinase; PDX = patient-derived xenograft; KRAS = Kirsten rat sarcoma viral oncogene homolog

#Includes gastric and colorectal cancers
*Includes head and neck SCC & colorectal cancer
+Includes colon, lung and breast cancer
